# Factors associated with neck and shoulder pain: a cross-sectional study among 16,000 adults in five county councils in Sweden

**DOI:** 10.1186/s12891-021-04753-0

**Published:** 2021-10-12

**Authors:** Gunnel Peterson, Nicklas Pihlström

**Affiliations:** 1 Centre for Clinical Research Sörmland, Uppsala University, SE- 631 88 Eskilstuna, Sweden; 2 Department of Health, Medicine and Caring Sciences, Physiotherapy, Linköping University, Linköping, Sweden

**Keywords:** Neck pain, Musculoskeletal pain, Shoulder pain, Epidemiology, Exercise, Health

## Abstract

**Background:**

Neck and shoulder pain is common in the general population, but studies on factors related to the risk of neck and shoulder pain have produced inconclusive results. Known factors related to pain include general physical activity, exercise, sleep disorders, and lifestyle, but further research is needed to improve our ability to prevent neck and shoulder pain. The aim was to investigate whether neck and shoulder pain are associated with physical domains (i.e., aerobic physical activities, general physical activities, and sitting time), sleep disturbances, general health, job satisfaction, and/or working time.

**Methods:**

This population-based, cross-sectional study was conducted in Sweden in 2017 and included 16,167 individuals, aged 18 to 63 years. We administered a questionnaire to determine neck and shoulder pain, the time spent in general physical activity or aerobic physical activity, the time spent sitting, sleep disturbances, general health, job satisfaction, and the time spent working. Factors associated with neck and shoulder pain were explored using logistic regression.

**Results:**

Significant factors associated with neck and shoulder pain were: overall health, sleep quality, and aerobic exercise. The odds of sustaining neck and shoulder pain increased with moderate or poor health (odds ratios [ORs]: 2.3 and 2.8, respectively) and sleep disorders (OR: 1.7). Conversely, aerobic physical activity performed more than 60 min/week at a level that enhanced respiratory and heart rate was associated with a reduced risk of experiencing neck and shoulder pain (OR: 0.8).

**Conclusions:**

Although no causal relationships could be determined in the present study, the results highlight important associations between aerobic exercise, undisturbed sleep, good health, and the absence of upper body pain. Exercises that enhance breathing and heart rate were associated with a reduced risk of experiencing neck or shoulder pain, but there was no association between general physical activity and upper body pain. Therefore, clinicians may not recommend low-intensity activities, such as walking, for preventing or improving neck and shoulder pain.

## Introduction

Musculoskeletal pain in the neck [1] and upper body are common [2], and they can affect the ability to work [3–5]. The one-month prevalence of neck pain in the world’s working population has been estimated at between 15.4 and 41.1% [1], and approximately 50% of these reported ongoing or recurrent pain [6, 7]. Combined musculoskeletal pain in the neck, upper body, and back affects both women (34%) and men (23%) [2]. Among men, high job strain was associated with upper body pain, but this association was not observed among women [2]. In contrast, Rashid et al. [8] reported that high pain intensity in the neck/shoulders and/or back and high job strain were associated with a low ability to work among women on sick leave [8]. However, in those studies [2, 8] physical activity and aerobic exercise habits were not analysed for a potential impact on the results. It is well known that regular physical activity can reduce the risks of premature mortality and disease development [9, 10]. Moreover, abundant evidence supports the notion that enhanced physical activity and exercise may be among the most important strategies for promoting good health [11].

However, studies investigating how physical activity and exercise are associated with neck pain and absence from work have provided inconclusive results [12–18]. An increase in regular walking reduced the onset of neck pain at work, but pain intensity and disability were not significantly different compared to a control group [18]. Frequent participation in sports activities was related to less frequent lower back pain, but not less frequent neck pain [14]. Hallman et al. [17] found that, among women, vigorous physical activity had a negative effect on the course of neck pain intensity. However, less-intense physical activities, like walking, running, or cycling, were associated with a lower intensity of neck pain [17]. Conversely, individuals with chronic neck pain displayed reduced participation in physical activities when the neck pain was associated with low pain thresholds and low pain tolerance [15]. Additionally, factors that influence neck and shoulder pain appear to be different between the sexes. Middle-aged women with high physical activity levels were more likely to recover from persistent neck pain [16] and had fewer sick-leave absences than women with low physical activity levels [13], but this was not true among middle-aged men [13, 16]. Although exercise therapy is often recommended for managing pain, exercise interventions aiming to reduce neck and shoulder pain and improve work ability have shown limited results [19–22]. Aerobic physical activity (i.e., activities that improve cardiorespiratory fitness), resistance training, and neck-specific exercises showed only small effects on neck and shoulder pain [19, 21] and work ability [20], compared to control groups. Despite these inconclusive findings on the impact of physical activity on neck and shoulder pain [13–21], it has been shown that inactivity, low fitness levels, and sitting during leisure time are related to more perceived neck and back pain [23]. Moreover, neck pain has been associated with sleep disturbances [24], and recovery from neck pain is less likely to occur in individuals with poor sleep quality, compared to individuals with good sleep quality [25].

Thus, although neck and shoulder pain can have an impact on work and leisure-time activities [17] by reducing the ability to engage in general physical activities and exercise, physical activity is crucial for good health, good sleep quality, general fitness, and physical functioning [11]. A sedentary lifestyle has been related to higher levels of pain and an elevated risk of contracting diseases, compared to an active lifestyle [11]. Therefore, it is important to gain more knowledge about how physical activity, the time spent sitting, work-related factors, and sleep disturbances affect pain in the neck and upper body.

The aim of this study was to investigate whether neck and shoulder pain is associated with physical domains (i.e., aerobic physical activities, general physical activities, and sitting time), sleep disturbances, general health, job satisfaction, and/or working time.

## Methods

This population-based, cross-sectional study, called Life and Health, was conducted in 55 municipalities from February to June 2017. The municipalities included approximately 1.2 million inhabitants in the middle region of Sweden. The study used an independent, random sample stratified according to age group, sex, and geographical data. A total of 78,000 individuals over 18 years old were randomly selected to participate in the survey. The survey comprised two questionnaires, one for younger adults (aged 18 to 30 years) that included 49 questions, and one for adults aged 31 years or older that included 62 questions. The questions covered different health-related topics, such as general health, quality of life, physical activity, smoking habits, dietary habits, alcohol consumption, socioeconomic factors, work, and social relationships. The questions included in the present study were identical in both questionnaires, and all measurements were self-reported. The Swedish report can be accessed at: https://samverkan.regionsormland.se/utveckling-och-samarbete/statistik/folkhalsoundersokningar/liv%2D%2Dhalsa/

### Study population

The study population included adults aged between 18 years and the age of retirement in Sweden (65 years). In total 34,313 (44%) individuals responded to the questionnaires. Of these, 18,286 (54%) individuals were between 18 and 64 years old and 15,847 (46%) were between 65 and 104 years. Of the 18,286 respondents, 16,167 individuals responded to the neck and shoulder pain question “Do you have pain in the neck or the shoulders?” and were included in the present study. Alternative responses to the question were: ‘No pain’, ‘Yes, some pain’, and ‘Yes, severe pain’.

### Potential predictive factors

Several potentially influential factors were investigated, based on their relevance and their previous identification in the literature as risk factors for neck and shoulder pain (Table 1). We investigated participants’ general state of health with the question: How do you rate your current general state of health? The potential responses were: ‘Very good’, ‘Good’, ‘Moderate’, ‘Poor’, or ‘Very poor’. We investigated sleep disorders with the question: Do you have problems sleeping? The potential responses were: ‘No’, ‘Yes, mild’, or ‘Yes, severe’.

**Table 1:** Baseline characteristics of the study population, grouped by the presence of neck pain (*n* = 16,167)

Characteristic	No neck/shoulder pain	Yes, neck/shoulder pain
	*N* = 8160 (50.5%)	*N* = 8007 (49.5%)
Men	4070 (60%)	2671 (40%)
Women	4090 (43%)	5336 (57%)
**Health status**^**a**^		
Very good	2523 (31%)	1051(13%)
Good	4365 (54%)	4063 (51%)
Moderate	1017 (12.5%)	2209 (28%)
Poor	183 (2%)	529 (7%)
Very poor	32 (0.5%)	116 (1%)
Total	8120	7968
**Sleep disturbances**		
No	6186 (73%)	4600 (58%)
Yes, mild	1703 (24%)	2552 (32%)
Yes, severe	243 (3%)	752 (10%)
Total	8132	7904
**Aerobic physical activity**^**b**^		
Less than 30 min/week	2840 (35%)	3481 (44%)
30 to 59 min/week	1282 (16%)	1331 (17%)
60 to 89 min/week	953 (12%)	955 (12%)
More than 90 min/week	3024 (37%)	2187 (27%)
Total	8099	7954
**General physical activity**^**c**^		
Less than 30 min/week	1012 (13%)	1080 (14%)
30 to 89 min/week	2694 (33%)	2681 (34%)
90 to 149 min/week	1387 (17%)	1304 (16%)
More than 150 min/week	3024 (37%)	2906 (36%)
Total	8117	7971
**Time spent sitting (per day)**		
More than 10 h	1327 (16%)	1356 (17%)
7 to 9 h	2257 (28%)	2032 (25%)
4 to 6 h	2992 (37%)	2995 (38%)
Less than 3 h	1524 (19%)	1562 (20%)
Total	8100	7945
**Socioeconomic status**		
Employed	5548 (63%)	4913 (59%)
Self-employed^d^	751 (8%)	662 (8%)
Leave of absence or parental leave	281 (3%)	286 (3%)
Studying	1504 (17%)	975 (11%)
Unemployed, sick-leave, pension	778 (9%)	1573 (19%)
Total^e^	8862	8409
**Job satisfaction**^**f**^		
Very high	2579 (42%)	2087 (34%)
High	2808 (46%)	2929 (48%)
Moderate	528 (8%)	556 (12%)
Low	205 (3.5%)	320 (5%)
None	17 (0.5%)	91 (1%)
Total	6151	6183
**Time spent working**		
≥ 21 h/week	482 (8%)	550 (9%)
≤ 20 h/week	5656 (92%)	5622 (91%)
Total	6138	6172

^a^Answer to the question: How do you rate your current general state of health?

^b^Aerobic physical activity was defined as exercises that enhanced breathing, for example running, aerobic exercises, or football

^c^General physical activity was defined as movement (e.g., walking, cycling, or work in the garden)

^d^Working as self-employed (e.g., businessman, entrepreneur)

^e^Participants in the study could select more than one answer for socio-economic status, e.g., partial sick leave and worked as employed

^f^Answer to the question: How would you rate your satisfaction with your current work?

General physical activity was defined as bodily movement generated by skeletal muscles (for example walking, cycling, or gardening) [11]. Aerobic physical activities were defined as aerobic exercise that enhanced the respiratory and heart rates (for example, running) at a moderate level [11]. Aerobic physical activities, general physical activities, and sitting times were self-reported with the following statements: 1) How much time per week do you spend in aerobic physical activities that enhance your breathing: for example, running, aerobic exercises, or football? The potential responses were: ‘0 min’, ‘less than 30 min’, ‘30–59 min (0.5 to 1 hr)’, ‘60–89 min (1 to 1.5 hrs)’, ‘90–119 min (1.5 to 2 hrs)’, or ‘2 hrs or more’. 2) How much time per week do you spend in general physical activities, for example walking, cycling, or working in the garden, during a normal week? Count all the time (minimum, 10 min per week). The potential responses were: ‘0 min’, ‘less than 30 min’, ‘30–59 min (0.5 to 1 h)’, ‘60–89 min (1 to 1.5 h)’, ‘90–149 min (1.5 to 2.5 hrs)’, ‘150–299 min (2.5 to 5 hrs)’, or ‘5 hrs or more’. 3) How much time do you spend sitting during a normal day? The potential responses were: ‘More than 12 hrs’, ‘10–12 h’, ‘7–9 h’, ‘4–6 h’, ‘1–3 h’, ‘less than 1 hrs’, or ‘sitting or lying more than 12 hrs per day, due to disability’.

Work-related factors were investigated with the question: How would you rate your satisfaction with your work situation? The potential responses were: ‘Very high’, ‘High’, ‘Moderate’, ‘Low’, or ‘None’. We also investigated the time spent working per week. The potential responses were: ‘20 hrs/week or less’ or ‘more than 21 hrs/week or more’.

Employment was evaluated with the question: What is your current occupation? The potential responses were: ‘working as an employee’, ‘self-employed (e.g., businessperson, entrepreneur)’, ‘leave of absence or on parental leave’, ‘study or practice’, ‘unemployed’, ‘pension’, ‘on sick leave’, or ‘others’.

The logistic regression also included the time spent working per week (20 h or less or more than 20 h) and socioeconomic status (employed, self-employed, leave of absence, parental leave, studying, unemployed, sick-leave, or pension).

### Statistics

We performed multivariate logistic regression analyses to estimate the associations between neck and shoulder pain and the variables. The responses to the question about neck or shoulder pain were dichotomised to distinguish between individuals with or without neck or shoulder pain. The variables were dichotomised as follows: Neck or shoulder pain: No/Yes (where Yes included the responses: ‘Yes, mild’ and ‘Yes, severe’); these groups distinguished between those with and without neck or shoulder pain. Variables that described general physical activity and aerobic physical activity were also dichotomised to distinguish between levels below or equal to/above the national recommendations current in 2017, which follow World Health Organisation recommendations [26]. Aerobic physical activities: < 60 min vs. ≥60 min; General physical activities: < 150 min vs. ≥150 min. Responses to the sitting time question were dichotomised to distinguish between individuals with a potential risk of enhanced risk for neck pain and individuals without this risk. The cut-off value for sitting time was based on Kallings et al., who showed that a sitting time of less than 7 h per day is associated with lower levels of neck and back pain [27]. The cut-off value for sitting time was thus < 7 h vs. ≥7 h. Responses to the work question were dichotomised to distinguish between individuals in work/study/partial leave and those outside the labour force: Yes (employed, self-employed, leave of absence, parental leave, or studying), vs. No (unemployed, sick-leave, or pension). The sleep question was dichotomised to distinguish individuals with and without sleep problems: No (no sleep problems) vs. Yes (‘Yes, mild’ and ‘Yes, severe’ sleep problems); and working hours per week: ≤20 h vs. > 20 h.

Two questions were trichotomised to distinguish between good, moderate, or poor/low health and job satisfaction: the health-related quality of life: good (= very good or good), moderate, and poor (= poor or very poor); and job satisfaction: high (= very high or high), moderate, and low (= low or none). Prior to carrying out the multivariate regression analysis, the crude odds ratio for each variable was calculated with respect to neck and shoulder pain to check for possible candidate variables. The candidate variables were then included in the multivariate regression analysis. Briefly, we performed a backwards, conditional, automatic variable selection, with *p*-value > 0.1 as removal criterion. The backwards selection stopped after four iterations, and three of the variables were removed from the final model. Significance levels were calculated using the Wald statistic, and the fitting of the proposed model was evaluated using the Nagelkerke R Square method. All analyses were performed using the SPSS 22.0 statistical package (IBM Corp., Armonk, N.Y., USA). Statistical significance was set at a level of *p* < 0.05.

## Results

The survey was sent to 78,000 individuals over 18 years old, and 34,313 individuals (44%) responded. Of these, 18,286 (54%) were 18 to 64 years old. Of these 18,286 respondents, 16,167 responded to the neck and shoulder pain question; thus, these respondents were included in the present study. The cohort included 9426 (58%) women and 6741 (42%) men, with a mean age of 41.5 years (SD: 14.4). Of the 16,167 participants, 8160 (50.5%) reported no neck or shoulder pain, and 8007 (49.5%) reported mild or severe neck or shoulder pain. A description of the study population is shown in Table 1. All results are based on self-reported outcomes.

### Predictors of neck and shoulder pain

The multivariate logistic regression revealed that current general health was significantly associated with neck and shoulder pain. Participants with self-reported moderate and poor-to-very poor current health had ORs of 2.3 and 2.8, respectively, for pain. In addition, neck and shoulder pain were associated with sleep disturbances, sitting for less than 7 h per day, and moderate or low job satisfaction. In contrast, aerobic physical activity for more than 60 min/week reduced the risk of neck pain (Table 2).

**Table 2:** Factors associated with neck and shoulder pain, final step of the multivariable regression model

Factor	B	S.E.	Wald	*P*-value	Odds ratio (95% CI)
Current health (Very good, Good)			296.066	<.001	
Current health (Moderate)	0.848	0.053	253.186	<.001	2.33 (2.10–2.60)
Current health (Poor, Very poor)	1.023	0.120	72.606	<.001	2.78 (2.20–3.52)
Sleep disturbance, Yes	0.526	0.043	148.471	<.001	1.69 (1.55–1.84)
Aerobic physical exercise (> 60 min/week)	−.226	0.038	34.575	<.001	0.80 (0.74–0.86)
Sitting time (≤6 h/day)	0.099	0.039	6.532	.011	1.10 (1.03–1.19)
Job satisfaction (Very high, High)			12.215	.002	
Job satisfaction (Moderate)	0.156	0.064	5.957	.015	1.17 (1.03–1.33)
Job satisfaction (Low, None)	0.246	0.090	7.453	.006	1.28 (1.07–1.53)
Constant	−0.327	0.039	71.092	<.001	0.721

Nagelkerke pseudo R2 = 0.09

## Discussion

The study revealed five factors that were significantly associated with neck and shoulder pain. General health showed the highest association with the risk of experiencing neck and shoulder pain. Participants with moderate or poor health were more than twice as likely (ORs: 2.3 and 2.8, respectively) as healthy individuals to experience neck and shoulder pain. Participants with sleep disturbances had an OR of 1.7 for neck and shoulder pain. We observed weak associations between neck and shoulder pain and poor job satisfaction (ORs: 1.2 to 1.3) and sitting for less than 7 h per day (OR: 1.1). Our results also showed that aerobic physical activity was negatively associated with neck and shoulder pain. Participants who performed aerobic exercise for more than 60 min per week, at a level that enhanced heart rate and breathing, were less likely to experience neck and shoulder pain (OR: 0.8) than participants who did not perform aerobic exercise. In this study, we distinguished between aerobic physical activity (physical activity that is planned, structured, and repetitive, which improves or maintains physical fitness) and general physical activity (any bodily movement generated by skeletal muscles that results in energy expenditure) [28]. For example, general physical activities included walking, cycling, or gardening.

Moderate or poor health showed the highest association with neck and shoulder pain. One of the most important ways to improve health in adults is to maintain active habits and to perform moderate physical activity for a minimum of 150 min per week and/or vigorous physical activity for 75 min per week. Moreover, additional health benefits can be gained with up to 5 h/week of moderate physical activity and 2.5 h/week of vigorous physical activity [11]. It is well known that physical activity and sedentary behaviour are associated with neck pain and poor health [11, 19, 23, 29]. However, there seem to be large variations in the combination of these variables among individuals [23]. Several health conditions have been shown to benefit from physical activity, for example, coronary heart disease, cancer, diabetes, and sleep disturbances [11]. However, in the current study, we found no significant association between > 150 min/week of general physical activity and risk of neck and shoulder pain. Overall, our participants reported low levels of general physical activity. Most participants performed general physical activities for less than 150 min/week (64% of participants with neck and shoulder pain and 63% of participants without pain). Office workers who reported walking as their general physical activity had a reduced incidence of neck pain onset, but there was no significant difference in neck pain intensity, compared to the control group [18]. In the present study, we found an association between aerobic physical activity, i.e., exercises that enhance breathing and heart rate, and lower odds of experiencing neck or shoulder pain. We found no association between physical activity (e.g., walking) and the risk of experiencing neck or shoulder pain. However, in the present study, we could not determine causal relationships; for example, a higher intensity of physical activity might have reduced pain in the upper body, or conversely, individuals with no neck pain may have been more likely to participate in moderate to vigorous exercise.

Despite the low levels of general physical activity in the current study, 85% of participants with no neck and shoulder pain reported good to very good health, compared to 64% of participants with neck and shoulder pain. However, the risk of developing a chronic condition, such as musculoskeletal disorders or pain, coronary heart disease, cancer, diabetes, or premature mortality, was likely to be considerable for many individuals, due to their low physical activity levels.

In contrast, more than 60 min/week of aerobic physical activity was associated with a reduced risk of neck and shoulder pain. Indeed, more than 60 min/week of aerobic exercise was reported by 49% of participants with no neck and shoulder pain, compared to 39% of individuals with neck and shoulder pain. In a previous study, physical activity for 5 h/week or more was associated with a lower risk of neck pain [30], but the intensity level was not explicitly explained in that study. Nevertheless, the authors showed that, for pain in the upper body, low-intensity physical activity (e.g., walking) may be insufficient to reduce pain intensity, when the activity is performed for less than 5 h/week [30]. Alternatively, as indicated in the present study, moderate aerobic physical activity might affect pain intensity. Currently, the available evidence for the effect of physical activity on neck and shoulder pain remains inconclusive [12, 16, 28, 29], but moderate-to-vigorous activity may be protective and can be included in clinical recommendations.

In the present study, neck and shoulder pain were associated with sleep disturbances. Previously, Holm et al. [4] found that the risk of developing neck and lower back pain was higher in individuals who reported poor work ability and sleep disturbances, and Finan et al. reported similar findings [31]. Individuals living with chronic pain experience sleep disturbances, due to the pain [32]. Our findings in the present study are consistent with those studies.

It has previously been reported that short periods of sitting are related to a lower risk of neck and back pain [23]. In contrast, in the present study, we found that sitting for less than 7 h per day was related to a small increase in the risk of neck and shoulder pain. However, too much activity, without rest during the day, may cause pain when the activity is static, repetitive [33], or related to stress [34]. Moreover, another study showed that, among blue-collar workers, increased sitting time at work reduced the risk of neck pain [35]. Thus, sitting and relaxation times, relative to physical demands on muscles, are likely to be highly individual.

We found that time spent working was not significantly related to neck and shoulder pain. However, we also found that neck and shoulder pain was weakly associated with poor job satisfaction.

### Study limitations

The main study limitation is that we could not draw causal relationships, due to the cross-sectional nature of our study. Another limitation is that self-reported estimates of exercise and activity levels have been questioned. For example, individuals with cancer or fibromyalgia have over-reported their physical activity levels in self-reported questionnaires [36, 37], and objective methods have been recommended. However, it would be extremely costly, and probably not possible, to measure physical activity objectively in all the individuals in a large, population-based cohort with several thousand participants. Moreover, self-reported and objective methods produced contradictory results in a study by Neupane et al. [38]. They found that musculoskeletal pain was significantly correlated with self-reported occupational physical activity, but not correlated with objectively measured activity levels. Therefore, other factors, apart from the fact that exercise and physical activity were self-reported, might have been associated with neck and shoulder pain. Two different questionnaires were used in the study. The questionnaire sent to individuals aged over 30 years included more questions than the one sent to individuals aged between 18 and 30 years. The reason for fewer questions being asked of the younger age group was to increase the response rate among younger adults. We do not think that this discrepancy between the questionnaires impacted the study results because the questions included in the present study were identical in both questionnaires. Another limitation relates to the question regarding neck or shoulder pain. This question had three response options: ‘No pain’, ‘Yes, mild pain’ or ‘Yes, severe pain’. There was no clear definition given to distinguish between mild and severe pain, and we did not use a validated pain measurement; all responses were based on subjective interpretations of the question and self-assessments. Individuals in the study could have experiences of pain in the neck and/or shoulders. The questionnaires included no questions about intensity, duration, or frequency of pain or a pain sketch. Thus, we could not draw any conclusions about potential associations between pain severity, pain location, and the predictive factors. However, the aim of this study was to distinguish between individuals with or without upper body pain (neck or shoulder pain) and to relate the presence/absence of this pain to potential explanatory factors. Moreover, only 14% of participants reported severe pain. Therefore, we considered it reasonable to forgo the distinction between mild and severe pain and dichotomise the responses into categories of no pain vs. pain. The question about disturbed sleep had limitations regarding information about the severity of sleeping problems. The question had only three response options: ‘No’, ‘Yes, mild sleep problems’, or ‘Yes, severe sleep problems’. However, the question was able to distinguish between individuals with and without sleep problems. We intended to select cut-off values that could distinguish between individuals who followed the current recommended physical activity level (in 2017, ≥150 min) [26] and/or aerobic physical activity level (≥75 min) [26] and individuals who practised activities below the recommended levels. Unfortunately, the response alternatives for aerobic exercise did not allow a threshold of 75 min. Therefore, we chose a 60-min cut-off, because it included all participants who followed the 75 min recommendation, and excluded most participants who did not fulfil the recommendation. To date, there is no clear recommendation regarding sitting time. Health risks and the potential impact on neck and shoulder pain that were related to sitting time appeared to be associated with exercise habits [39]. Moreover, sitting times of > 6–8 h appeared to be related to an increased risk of disease and an enhanced risk of experiencing neck and back pain [27]. Therefore, we chose a cut-off of 7 h. Dichotomising and trichotomising variables had some potential limitations. Firstly, they might lead to an underestimation of the extent of variation in the outcomes. Secondly, individuals close to the cut-off value were considered to be very different from individuals who were just on the opposite side of the cut-off value, but in fact, they might be very similar.

In the present study, the dichotomisation for neck pain distinguished between individuals who expressed a ‘No’ response and individuals who expressed one of two responses that indicated a ‘Yes’ response. Therefore, we could clearly distinguish between individuals with and without neck/shoulder pain, individuals with and without sleep disorders, and individuals who followed or did not follow the current recommendations for physical activity and aerobic exercise. However, for two questions, health and job satisfaction, the responses could not be easily dichotomised. The central value (moderate) could have referred to either ‘moderately good’ or ‘moderately poor’ response alternatives. Therefore, we could not readily assign the ‘moderate’ category to either ‘good’ or ‘poor’. Instead, we decided to trichotomise the responses to distinguish between good, moderate, and poor/low.

## Conclusion

This study found that poor health and sleep disturbances were associated with neck or shoulder pain. In contrast, exercises that enhance breathing and heart rate reduced the risk of experiencing pain. This understanding of the association between upper body pain, aerobic exercise, sleep, and health may provide insights into preventive strategies and treatments for neck and shoulder pain. Our results show no association between general physical activity and the risk of upper body pain. Therefore, it may not be useful to recommend low-intensity activities, such as walking, for preventing or improving neck and shoulder pain. These results can be generalised to individuals between the ages of 18 and 64 years. Longitudinal studies are needed to gain a better understanding of how neck and shoulder pain, health, sleep disturbances, and aerobic exercise are related.

## Data Availability

Due to ethical restrictions, the authors had to seek permission to use the data for this study. Data is available from the corresponding author upon reasonable request.

## References

[1] Fejer R, Kyvik KO, Hartvigsen J (2006). The prevalence of neck pain in the world population: a systematic critical review of the literature. Eur Spine J.

[2] Sommer TG, Frost P, Svendsen SW (2015). Combined musculoskeletal pain in the upper and lower body: associations with occupational mechanical and psychosocial exposures. Int Arch Occup Environ Health.

[3] Jun D, Zoe M, Johnston V, O'Leary S (2017). Physical risk factors for developing non-specific neck pain in office workers: a systematic review and meta-analysis. Int Arch Occup Environ Health.

[4] Holm LW, Bohman T, Lekander M, Magnusson C, Skillgate E (2020). Risk of transition from occasional neck/back pain to long-duration activity limiting neck/back pain: a cohort study on the influence of poor work ability and sleep disturbances in the working population in Stockholm County. BMJ Open.

[5] Hallman DM, Rasmussen CDN, Jorgensen MB, Holtermann A (2018). Time course of neck-shoulder pain among workers: a longitudinal latent class growth analysis. Scand J Work Environ Health.

[6] Vos CJ, Verhagen AP, Passchier J, Koes BW (2008). Clinical course and prognostic factors in acute neck pain: an inception cohort study in general practice. Pain Med.

[7] Vasseljen O, Woodhouse A, Bjorngaard JH, Leivseth L (2013). Natural course of acute neck and low back pain in the general population: the HUNT study. Pain..

[8] Rashid M, Kristofferzon ML, Heiden M, Nilsson A (2018). Factors related to work ability and well-being among women on sick leave due to long-term pain in the neck/shoulders and/or back: a cross-sectional study. BMC Public Health.

[9] Professional Associations for Physical Activity, Sweden (Yrkesföreningar för fysisk aktivitet, YFA). Physical activity in the prevention and treatment of disease. Stockholm: Swedish National Institute of Public Health; 2010.

[10] Piercy KL, Troiano RP, Ballard RM, Carlson SA, Fulton JE, Galuska DA (2018). The physical activity guidelines for Americans. Jama..

[11] Bull FC, Al-Ansari SS, Biddle S, Borodulin K, Buman MP, Cardon G (2020). World Health Organization 2020 guidelines on physical activity and sedentary behaviour. Br J Sports Med.

[12] Sitthipornvorakul E, Janwantanakul P, Purepong N, Pensri P, van der Beek AJ (2011). The association between physical activity and neck and low back pain: a systematic review. Eur Spine J.

[13] Lopez-Bueno R, Calatayud J, Lopez-Sanchez GF, Smith L, Andersen LL, Casajus JA (2020). Higher leisure-time physical activity is associated with lower sickness absence: cross-sectional analysis among the general workforce. J Sports Med Phys Fitness.

[14] Kaartinen S, Aaltonen S, Korhonen T, Latvala A, Mikkelsson M, Kujala UM (2019). Is diversity of leisure-time sport activities associated with low back and neck-shoulder region pain? A Finnish twin cohort study. Prev Med Rep.

[15] Mansfield M, Thacker M, Spahr N, Smith T (2018). Factors associated with physical activity participation in adults with chronic cervical spine pain: a systematic review. Physiotherapy..

[16] Rasmussen-Barr E, Bohman T, Hallqvist J, Holm LW, Skillgate E (2013). Do physical activity level and body mass index predict recovery from persistent neck pain in men and women of working age? A population-based cohort study. Eur Spine J.

[17] Hallman DM, Birk Jorgensen M, Holtermann A (2017). Objectively measured physical activity and 12-month trajectories of neck-shoulder pain in workers: a prospective study in DPHACTO. Scand J Public Health.

[18] Sitthipornvorakul E, Sihawong R, Waongenngarm P, Janwantanakul P (2020). The effects of walking intervention on preventing neck pain in office workers: a randomized controlled trial. J Occup Health.

[19] Blangsted AK, Sogaard K, Hansen EA, Hannerz H, Sjogaard G (2008). One-year randomized controlled trial with different physical-activity programs to reduce musculoskeletal symptoms in the neck and shoulders among office workers. Scand J Work Environ Health.

[20] Ting JZR, Chen X, Johnston V. Workplace-based exercise intervention improves work ability in office workers: a cluster randomised controlled trial. Int J Environ Res Public Health. 2019;16(15):2633. 10.3390/ijerph16152633 PMC6696298 31344787

[21] Pereira M, Comans T, Sjogaard G, Straker L, Melloh M, O'Leary S (2019). The impact of workplace ergonomics and neck-specific exercise versus ergonomics and health promotion interventions on office worker productivity: a cluster-randomized trial. Scand J Work Environ Health.

[22] Forsbrand MH, Turkiewicz A, Petersson IF, Sennehed CP, Stigmar K (2020). Long-term effects on function, health-related quality of life and work ability after structured physiotherapy including a workplace intervention. A secondary analysis of a randomised controlled trial (WorkUp) in primary care for patients with neck and/or back pain. Scand J Prim Health Care.

[23] Ekblom-Bak E, Stenling A, Salier Eriksson J, Hemmingsson E, Kallings LV, Andersson G (2020). Latent profile analysis patterns of exercise, sitting and fitness in adults - associations with metabolic risk factors, perceived health, and perceived symptoms. PLoS One.

[24] Scarabottolo CC, Pinto RZ, Oliveira CB, Tebar WR, Saraiva BTC, Morelhao PK (2020). Back and neck pain and poor sleep quality in adolescents are associated even after controlling for confounding factors: an epidemiological study. Sleep Sci.

[25] Kovacs FM, Seco J, Royuela A, Melis S, Sanchez C, Diaz-Arribas MJ (2015). Patients with neck pain are less likely to improve if they experience poor sleep quality: a prospective study in routine practice. Clin J Pain.

[26] World Health Organization. Global Recommendations on Physical Activity for Health. Geneva: World Health Organization, 2010. http://whqlibdoc.who.int/publications/2010/9789241599979_eng.pdf?ua=1 (29 Aug 2021, date last accessed). 26180873

[27] Kallings LV, Blom V, Ekblom B, Holmlund T, Eriksson JS, Andersson G (2021). Workplace sitting is associated with self-reported general health and back/neck pain: a cross-sectional analysis in 44,978 employees. BMC Public Health.

[28] Caspersen CJ, Powell KE, Christenson GM (1985). Physical activity, exercise, and physical fitness: definitions and distinctions for health-related research. Public Health Rep.

[29] Bohman T, Holm LW, Hallqvist J, Pico-Espinosa OJ, Skillgate E (2019). Healthy lifestyle behaviour and risk of long-duration troublesome neck pain among men and women with occasional neck pain: results from the Stockholm public health cohort. BMJ Open.

[30] Kirsch Micheletti J, Blafoss R, Sundstrup E, Bay H, Pastre CM, Andersen LL (2019). Association between lifestyle and musculoskeletal pain: cross-sectional study among 10,000 adults from the general working population. BMC Musculoskelet Disord.

[31] Finan PH, Goodin BR, Smith MT (2013). The association of sleep and pain: an update and a path forward. J Pain.

[32] Mathias JL, Cant ML, Burke ALJ (2018). Sleep disturbances and sleep disorders in adults living with chronic pain: a meta-analysis. Sleep Med.

[33] Sogaard K, Sjogaard G (2017). Physical activity as cause and cure of muscular pain: evidence of underlying mechanisms. Exerc Sport Sci Rev.

[34] Fanavoll R, Nilsen TI, Holtermann A, Mork PJ (2016). Psychosocial work stress, leisure time physical exercise and the risk of chronic pain in the neck/shoulders: longitudinal data from the Norwegian HUNT study. Int J Occup Med Environ Health.

[35] Overas CK, Villumsen M, Axen I, Cabrita M, Leboeuf-Yde C, Hartvigsen J (2020). Association between objectively measured physical behaviour and neck- and/or low back pain: a systematic review. Eur J Pain.

[36] Wagoner CW, Choi SK, Deal AM, Lee JT, Wood WA, Muss HB (2019). Establishing physical activity in breast cancer: self-report versus activity tracker. Breast Cancer Res Treat.

[37] Benitez-Porres J, Delgado M, Ruiz JR (2013). Comparison of physical activity estimates using international physical activity questionnaire (IPAQ) and accelerometry in fibromyalgia patients: the Al-Andalus study. J Sports Sci.

[38] Neupane S, Karstad K, Hallman DM, Rugulies R, Holtermann A (2020). Objectively measured versus self-reported occupational physical activity and multisite musculoskeletal pain: a prospective follow-up study at 20 nursing homes in Denmark. Int Arch Occup Environ Health.

[39] Stamatakis E, Gale J, Bauman A, Ekelund U, Hamer M, Ding D (2019). Sitting time, physical activity, and risk of mortality in adults. J Am Coll Cardiol.

